# Developmental Patterns in Cooperative Contexts: How Children Integrate Social Strategy and Resource Endowment in Third-Party Responses

**DOI:** 10.3390/bs16081422

**Published:** 2026-08-19

**Authors:** Xuefei Pan, Zonghua Shi, Xinrong Guo, Xinxia Liu, Yanjie Su

**Affiliations:** 1 School of Psychological and Cognitive Sciences, Peking University, Beijing 100871, China; pxfyuhe@pku.edu.cn (X.P.); 17864231820@163.com (X.G.); 2401220028@stu.pku.edu.cn (X.L.); 2 Beijing Key Laboratory of Brain-Computer Interface and Mental Health Modulation, Beijing 100871, China; 3 Key Laboratory of Machine Perception (Ministry of Education), Peking University, Beijing 100871, China; 4 Department of Applied Psychology, Steinhardt School of Culture, Education, and Human Development, New York University, New York, NY 10003, USA; zs3301@nyu.edu

**Keywords:** cooperation, self-reliance, free-riding, third-party responses, resource endowment, fairness, social reasoning, moral development

## Abstract

This cross-sectional study examined how children integrated social strategy and initial resource endowment when responding to others as third-party observers in a cooperative context. Using an age-appropriate collective dilemma game, 141 Chinese children observed fictional peers choosing among cooperation, self-reliance, and free-riding strategies under conditions of high or low resource endowment, yielding a 3 (strategy) × 2 (resource endowment) experimental design. Children responded to each target through punishment, social evaluation, and partner choice. Results showed a strong preference for high-resource individuals among younger children (ages 5–6), which diminished with age, suggesting a developmental shift away from reliance on external cues such as resource. Across all age groups, children consistently responded most negatively to free riders, regardless of resource endowment, indicating their moral sensitivity to exploitative intent and fairness violations. Importantly, self-reliant individuals with high resources were viewed more favorably than those with low resources, indicating that children adjust their responses based on social strategy and resource endowment. These findings suggest two key developmental patterns: (1) an age-related decline in resource-based preferences and (2) early-emerging ability that enables children to weigh contextual cues when responding to non-cooperative behaviors. The study highlights age-related patterns in children’s third-party responses, reflecting their sensitivity to both social strategy and situational information in cooperative contexts.

## 1. Introduction

Cooperation has been a key social strategy for addressing complex social problems throughout human history (Van Lange et al., 2015). Cooperation enables more efficient resource management and payoff distribution, thereby enhancing collective productivity (Clemons & Row, 1992). Through cooperation, group members can pool their individual capacities, share benefits, and achieve goals that would otherwise be unattainable individually (Henrich & Muthukrishna, 2021). From an evolutionary perspective, cooperation serves as an important survival strategy by increasing group adaptability in challenging environments and maximizing cost-effectiveness to improve life quality (Kenward et al., 2015; Tomasello, 2009).

Despite these advantages, non-cooperative strategies remain prevalent in real-world contexts. Prior research has identified two distinct forms of non-cooperation: free-riding and self-reliance. Free-riding refers to the speculative behavior in which individuals exploit the benefits gained from collective solutions without contributing their fair share of efforts (Olson, 1971; Sweeney, 1973). Free-riding detriments cooperation by directly placing extra burden on cooperators to compensate for free riders (Kerr & Bruun, 1983). In contrast, self-reliance, while less studied, can also be detrimental to cooperation outcomes (Gross et al., 2020; Gross & Böhm, 2020; Gross & De Dreu, 2019). Self-reliance refers to a behavior in which individuals use their own resources to solve a shared problem privately rather than contributing to the collective solution (Gross & De Dreu, 2019). Unlike autonomy, independence, and individualism, which refer more broadly to self-directed orientations or tendencies, self-reliance is a non-cooperative strategy rather than a general personal characteristic. This strategy is not limited to laboratory dilemmas but arises frequently in real-world contexts, where individuals may choose private solutions instead of participating in coordinated group efforts. For example, in addressing climate change, countries may cooperate through international efforts to reduce emissions, or rely primarily on their own resources and measures to cope with climate-related risks. Such self-reliant choices undermine group outcomes by causing inefficiency due to repeated individual efforts and resource waste, therefore foregoing the benefits of economies of scale (Gross et al., 2020). Thus, self-reliance represents a theoretically meaningful form of non-cooperation, providing a useful basis for examining whether individuals distinguish between different ways of disengaging from collective cooperation.

However, free-riding and self-reliance differ in several important ways. Free riders intentionally benefit from others’ efforts without contributing themselves, thus deliberately undermining cooperation through malicious exploitation. This behavior violates perceived fairness, a concept that children are highly sensitive to from a young age and that drives social intervention (Blake et al., 2015; Engelmann & Tomasello, 2019; Fehr et al., 2008; Kenward & Dahl, 2011; Lee & Warneken, 2022; Schmidt & Sommerville, 2011). As a result, children may be more likely to respond negatively and intervene when they observe free-riding. On the other hand, self-reliance does not reflect this exploitative intent. Often, self-reliant individuals opt out of cooperation due to factors such as risk aversion, low trust in group members, or a preference for individual work (Gross et al., 2020; Gross & Böhm, 2020; Gross & De Dreu, 2019; Malthouse et al., 2026). Moreover, because self-reliant individuals pursue independent solutions by exerting comparable individual effort, their actions are less likely to violate perceptions of fairness (Gross & Böhm, 2020).

The two types of noncooperative behaviors differ in terms of intentionality and fairness violations, which may shape children’s third-party perceptions, leading to divergent patterns of social responses. Research on moral development suggests that children are sensitive to morally relevant features of social behavior from an early age. For example, preschool children distinguish moral transgressions from conventional violations and judge the former as more serious and deserving of punishment (Smetana, 1981). Importantly, children’s social judgments become more sensitive to actors’ mental states across development. Between the ages of four and eight, children increasingly incorporate intentions, rather than relying primarily on outcomes, when making moral judgments (Cushman et al., 2013). Together, these findings suggest that children’s third-party responses may reflect the developing integration of observable behavior, inferred intentions and social characteristics. This developmental perspective is particularly relevant to free-riding and self-reliance, because children may respond differently to these strategies based on the intentions and social characteristics they infer from them.

Beyond these strategy-related considerations, children’s third-party responses may also be shaped by the social context in which the behaviors occur (Gross et al., 2020). One critical contextual factor that can affect both players’ strategic decisions and third-party interpretations is resource endowment, which describes the differences in initial resource distribution among group members, thus influencing the extent to which individuals have to depend on group efforts to achieve collective solutions. Resource endowment can directly affect social strategy: individuals with ample resources can easily solve problems independently at an acceptable cost, while individuals less affluent may face a great cost and thus are more dependent on group cooperation. Gross et al. (2020) explored the role of asymmetric resource endowment in shaping cooperation strategy. They found that group members with greater resources were more likely to pursue self-reliant strategies compared to those with fewer resources, suggesting that resource endowment influences individuals’ strategy choices in social dilemmas. However, because their study was conducted with participants as players within the cooperation game, it remains unclear how resource endowment influences responses from a third-party perspective. In third-party scenarios, people are not required to engage in strategic gameplay, and thus self-interest is not directly involved; instead, they adopt an evaluative stance toward others’ behavior. This allows the effect of resource endowment to be examined as a social cue, thereby offering valuable insight into social perceptions and behaviors.

Despite this potential, to the best of our knowledge, little research has examined how resource endowment influences children’s third-party responses in cooperative contexts. Existing studies show that, by age four, children are sensitive to differences in resource endowment and may use such information as one cue when reasoning about others’ social status (Heck et al., 2022). Meanwhile, young children tend to prefer individuals with more resources (Heck et al., 2022). This preference tends to diminish with age as children develop more sophisticated social evaluation (Shu et al., 2024; X. Yang & Dunham, 2022). However, it remains unclear how children balance contextual cues like resource endowment with cooperative behavioral strategies when forming social responses. Notably, Kenward et al. (2015) found that four-year-old children strategically favored wealthier recipients only when those recipients had stated an intention to reciprocate. Similarly, Xiong et al. (2016) showed that children shared more with recipients who had the potential to reciprocate, particularly when those recipients possessed valuable resources. These findings suggest that even at a young age, children integrate both resource-related information and reciprocity expectations when making allocation decisions. These patterns mark an early-developing capacity for strategic social reasoning that goes beyond surface-level preferences.

Hence, it is essential to take a developmental approach to understand children’s increasing complexity and flexibility in making context-sensitive social responses. Examining how children integrate contextual cues to respond to different strategies in cooperation can help identify developmental trajectories in forming social reasoning. This approach may also identify potential sensitive periods and developmental patterns, offering practical implications for supporting children’s social and moral development.

Our study investigated how children aged 5–10 respond to cooperation decisions as third-party observers when provided with information about resource endowment. Following prior studies, we measured their social responses through three dimensions: evaluation, partner choice, and punishment (Chudek & Henrich, 2011; Warneken, 2018; Marshall et al., 2024; Marshall & McAuliffe, 2022; Schmidt & Rakoczy, 2023). We generally hypothesized that children would demonstrate a stronger preference towards high resource endowment targets compared to low resource endowment targets across different dimensions of social responses, and this tendency would diminish with age. More importantly, we hypothesized an interaction between resource endowment and social strategy: specifically, that self-reliance and cooperation would be viewed as more acceptable when resources were abundant, whereas the acceptability of free-riding would remain unaffected. This prediction is grounded in the inherent differences among these strategies. Unlike free-riding, which directly exploits collective efforts without contributing, self-reliance does not impose costs on others; thus, individuals may be more tolerant of self-reliance when resources are abundant. Similarly, resource endowment may also shape responses to cooperators. Because cooperation benefits the group, high-resource cooperators may be perceived as having a greater capacity to contribute and, consequently, as being more likely to promote collective success than low-resource cooperators.

## 2. Methods

### 2.1. Participants

We conducted a prior power analysis for the between-subjects effect of age group in a mixed-design F test (ANOVA: Repeated measures, between factors) using G*Power version 3.1 (Heinrich Heine University Düsseldorf, Düsseldorf, Germany) (Faul et al., 2007, 2009). The analysis specified three age groups and six repeated measurements. A correlation of 0.50 among the repeated measures was assumed. With f = 0.25, α = 0.05 and 1 − β = 0.90, the sample size required was 123. Participants were recruited through an online poster posted on the laboratory’s official WeChat public account. Parents registered voluntarily and could share the recruitment information with other parents through their networks. To ensure that the final analytic sample would remain adequately powered after allowing for incomplete participation or exclusions due to task comprehension, we recruited 150 children. Two children did not complete the task, and seven additional children were excluded because they could not adequately understand the rules or strategies of the task. The final analytic sample comprised 141 participants (M_age_ = 8.03, SD_age_ = 1.65, 80 girls). 45 children were aged 5–6 (29 girls), 48 children were aged 7–8 (25 girls), and 48 children were aged 9–10 (26 girls). All participants were Han Chinese children attending public kindergartens or primary schools in Beijing. Children’s socioeconomic background was not directly measured because testing took place within a relatively limited data-collection time. All children and their guardians consented to participate in the study. All children were tested in a campus laboratory. All children had normal or corrected-to-normal vision, with no history of color blindness, color weakness, psychiatric disorders, neurological conditions, cognitive impairments, chronic pain, or substance abuse. All procedures were approved by the ethics committee at Peking University.

### 2.2. Design and Procedure

The study employed a 3 (Age: 5–6, 7–8, 9–10 years) × 3 (Target: free rider, self-reliant member, cooperator) × 2 (Resource endowment: low, high) mixed design, with Age as a between-subject factor, Target and Resource endowment as within-subject factors. The Age variable was used to categorize participants into three distinct age groups. Target referred to players who chose free-riding, self-reliance, or cooperation in the collective dilemma game. Resource endowment was manipulated through the initial number of tokens assigned to the observed target. Low resource endowment targets had four tokens, and high resource endowment targets had eight tokens at the start of the game. The experimental task was presented to participants using PowerPoint (Microsoft Corporation, Redmond, WA, USA) slides featuring picture stimuli, accompanied by verbal instructions from the experimenter. The study underwent six rounds (3 types of target × 2 levels of resource endowment). The order of conditions was counterbalanced across participants.

Upon each participant’s arrival, the experimenter explained the overall rules of the collective dilemma game and the three possible strategies: cooperation, self-reliance, and free-riding. Building on the threshold public goods game used in prior adult cooperation research (Gross & De Dreu, 2019; Gross et al., 2020), we developed the collective dilemma game as an age-appropriate version for children in middle childhood. The experimental scenarios and visual materials were preliminarily evaluated and refined before formal data collection to ensure their clarity and suitability for children. In the game, children were placed in four-member groups, each starting with a fixed number of tokens placed on a shared lawn. Mice on the lawn would attempt to steal the players’ tokens. To protect their tokens from being stolen, each player could choose one of three strategies: (1) self-reliance, by spending three tokens to build a small fence that could protect only their own tokens, (2) cooperation, by contributing toward a total of eight tokens to build a large fence that would safeguard the tokens of all players, or (3) free-riding: by contributing nothing while relying on others to build the large fence. If no fence was built, all remaining tokens would be stolen. The game was designed to reflect an intermediate level of group interdependence (Gross & Böhm, 2020): if all players chose self-reliance, the group would spend a total of 12 tokens (three tokens per player) to protect everyone’s tokens. In contrast, if all players chose cooperation, the group could achieve the same outcome by only spending eight tokens (on average, two tokens per player). This setup reflected real-world situations in which individual solutions are possible, but cooperative solutions are often more cost-efficient. See Figures S1 and S2 for details of the game. After the experimenter had presented the game rules, checks were conducted to assess the children’s comprehension of the rules and strategies. If a child failed to understand a particular rule or strategy, the experimenter re-explained the relevant content. If children failed to comprehend after two rounds of explanation, they were excluded from the study.

Next, children were asked to observe the choices of other same-gender peers (targets) as third-party observers in the collective dilemma game. There were three types of targets: cooperator, self-reliant member, and free rider. The experimenter presented the target’s initial resource to the children and then informed them of the choice of this target in the game. Importantly, each target was presented to children individually and described as coming from a different four-person group, such that the choices of other group members were not disclosed. Children were told that the game comprised several rounds and that the tokens earned would be exchanged for gifts at the end. Targets who earned more tokens would receive eight gifts, whereas those who earned fewer tokens would receive four. The reward thresholds were deliberately withheld, and token earnings were kept relatively low so that all targets in the third-party scenario ultimately received the same payoff (four gifts). By holding final payoffs constant across targets, this design allowed us to isolate children’s responses to the strategies themselves while excluding potential confounds arising from differences in outcomes.

Before responding, children recalled the target’s initial token endowment. If incorrect, the information was repeated and the question was asked again. Then, the experimenter measured children’s responses through three aspects: punishment (“Do you want to take back the child’s gifts?”), evaluation (“What kind of child do you think he/she is?”), and partner choice (“If possible, do you want to partner with this child?”). For the punishment item, children were asked how many gifts they would like to take back, with larger numbers indicating a stronger punitive response. The experimenter explained what “taking back gifts” meant and checked for comprehension before the children’s response. Punishment scores ranged from 0 to 4, with higher scores indicating stronger punishment. Evaluation and partner choice questions were presented using a 5-point Likert scale adapted in cartoon with predefined symbols (Li et al., 2019; Wang et al., 2024). For the evaluation item, response options ranged from “a very bad child” to “a very good child” (−2 to 2), with higher scores indicating more positive evaluations. For the partner choice item, response options ranged from “definitely do not hope” to “definitely hope” (−2 to 2), with higher scores indicating a stronger preference to choose the target as a partner, which was intended to capture children’s general preference for context-independent partnership. For both items, the experimenter explained the meaning of each symbol to ensure comprehension. These task-based measures were adapted from commonly used approaches in developmental research on children’s sociomoral evaluation, punishment, and social preference (Lee & Warneken, 2020; Martin et al., 2022; Wang et al., 2024). At the end of the study, each child received several gifts for participating and was debriefed that all players were fictional.

## 3. Results

Children were grouped into three age ranges (5–6, 7–8, and 9–10 years) to facilitate comparisons across different periods of childhood, maintain relatively comparable sample sizes across groups, and remain consistent with the age-group structure specified in the prior power analysis. A more detailed rationale for this grouping is provided in the Supplementary Materials (see the “Age Grouping” section).

We used R 4.5.1 (R Foundation for Statistical Computing, Vienna, Austria) (R Core Team, 2025) with afex (Singmann et al., 2024) and emmeans (Lenth, 2024) packages for data analysis. To examine the influence of different factors on children’s social responses, we conducted 3 (Age: 5–6, 7–8, and 9–10 years) × 3 (Target: free rider, self-reliant member, cooperator) × 2 (Resource endowment: low, high) repeated-measures ANOVA. Punishment, evaluation, and partner choice were treated as three prespecified and conceptually distinct dependent variables and were analyzed using separate ANOVAs. Mauchly’s test was used to assess the sphericity assumption, and Greenhouse–Geisser corrections were applied when sphericity was violated. For significant effects requiring follow-up comparisons, Tukey’s HSD procedure was used to adjust for multiple pairwise comparisons. Complete descriptive statistics for all combinations of age group, target, and resource endowment across the three dependent variables are provided in Table S1 of the Supplementary Materials.

### 3.1. Punishment

**Figure 1:**
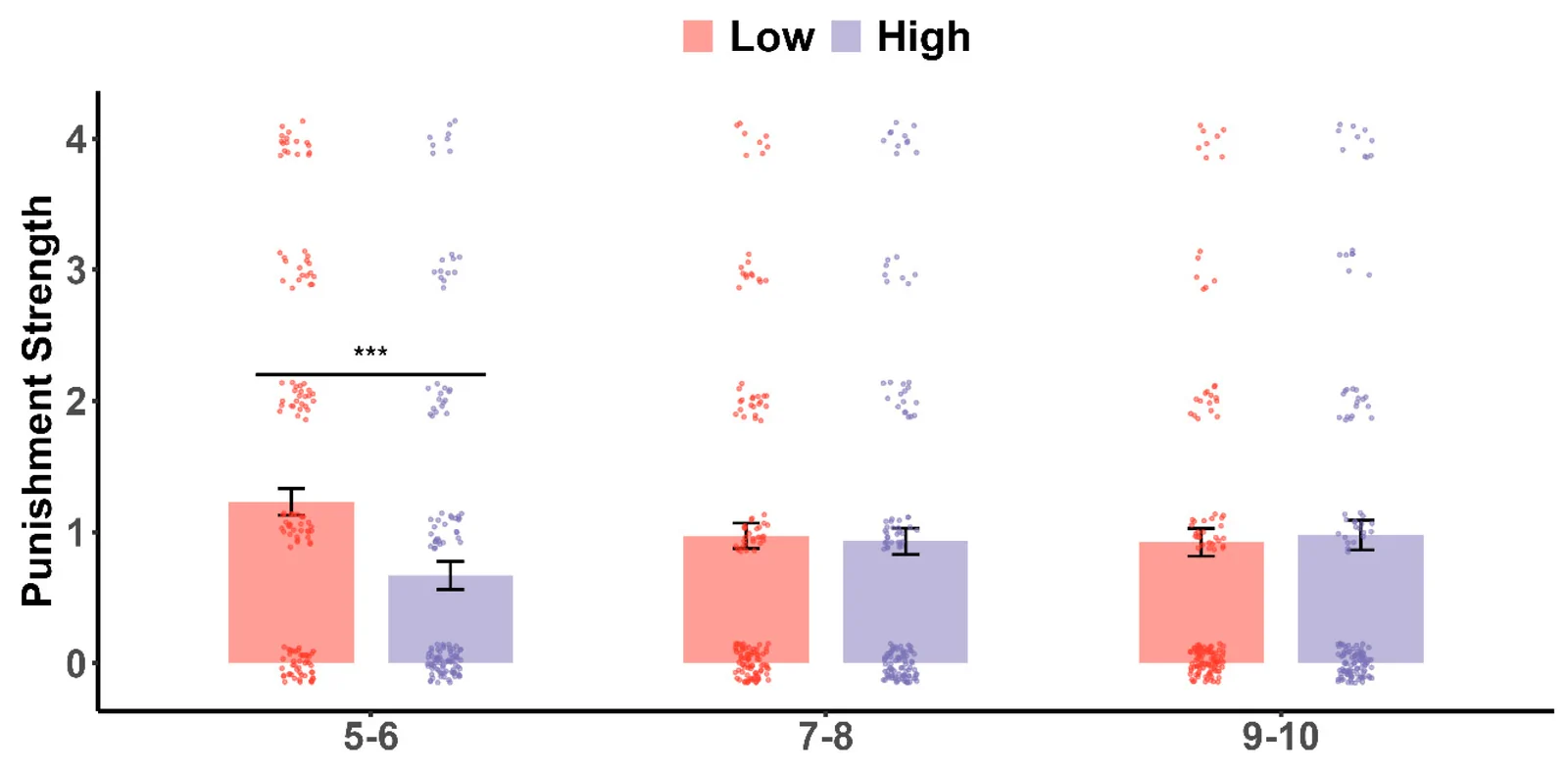
Interaction effects of different age and resource endowment on children’s punishment patterns. Error bars represent the ±1 standard error of the mean. *** *p* < 0.001. Scatter points represent the data of each child with jitters. Asterisks indicate significant differences between the high- and low-resource-endowment conditions within each age group.

**Figure 2:**
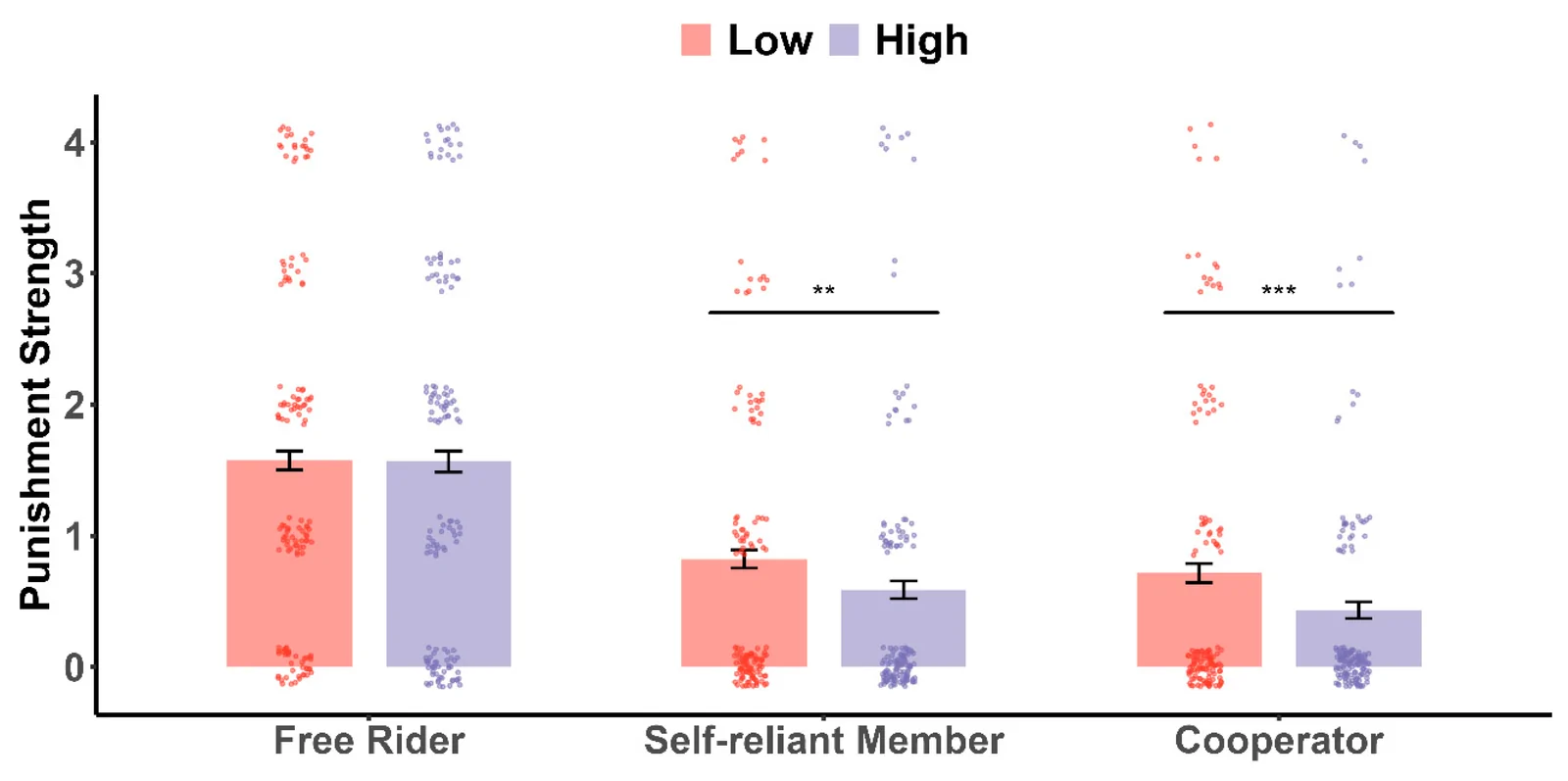
Interaction effects of different targets and resource endowment on children’s punishment patterns. Error bars represent the ±1 standard error of the mean. ** *p* < 0.01, *** *p* < 0.001. Scatter points represent the data of each child with jitters. Asterisks indicate significant differences between the high- and low-resource-endowment conditions within each target type.

There was a significant interaction between age and resource endowment (Figure 1), F_(2, 138)_ = 15.90, *p* < 0.001, η^2^_partial_ = 0.19, such that children aged 5–6 punished high-resource target (Mean = 0.90, 95% CI = [0.64, 1.17]) less compared to low-resource target (Mean = 1.47, 95% CI = [1.18, 1.76]), while children aged 7–8 (High: Mean = 0.90, 95% CI = [0.64, 1.15]; Low: Mean = 0.94, 95% CI = [0.66, 1.22]) and 9–10 (High: Mean = 0.79, 95% CI = [0.53, 1.05]; Low: Mean = 0.74, 95% CI = [0.46, 1.02]) did not show a significant difference. We also found a significant interaction between target and resource endowment (Figure 2), F_(1.91, 263.26)_ = 4.09, *p* < 0.05, η^2^_partial_ = 0.03. Children punished self-reliant members with high resource endowment (Mean = 0.59, 95% CI = [0.41, 0.76]) less compared to those with low resource endowment (Mean = 0.83, 95% CI = [0.63, 1.03]). Similarly, children punished cooperators with high resource endowment (Mean = 0.44, 95% CI = [0.29, 0.59]) less compared to those with low resource endowment (Mean = 0.73, 95% CI = [0.55, 0.90]). However, punishment levels were consistently high for free riders (High: Mean = 1.57, 95% CI = [1.33, 1.80]; Low: Mean = 1.58, 95% CI = [1.36, 1.81]), regardless of their resource endowment. Additionally, there was a main effect of resource endowment, F_(1, 138)_ = 14.74, *p* < 0.001, η^2^_partial_ = 0.10, such that children punished targets with high resource endowment (Mean = 0.86, 95% CI = [0.71, 1.01]) significantly less than targets with low resource endowment (Mean = 1.05, 95% CI = [0.88, 1.21]).

There was also a significant interaction between age and target (F_(3.91, 269.94)_ = 2.66, *p* < 0.05, η^2^_partial_ = 0.04) and a significant main effect of target (F_(1.96, 269.94)_ = 75.61, *p* < 0.001, η^2^_partial_ = 0.35). These effects replicate findings from a separate study using an independent sample. However, these results fall outside the primary scope of the present study. Hence, we report the principal statistical outcomes for transparency but do not further elaborate on the detailed patterns or discuss their theoretical implications in the present article. The same reporting approach is applied to the corresponding effects for evaluation and partner choice below. Briefly, the interaction findings showed that children aged 5–6 (*p* = 0.59) and 7–8 (*p* = 0.66) did not significantly differ in their punishment of self-reliant individuals and cooperators; however, children aged 9–10 punished self-reliant individuals significantly more than cooperators (*p* < 0.05). Moreover, children aged 5–6, 7–8, and 9–10 punished self-reliant individuals or cooperators significantly less than free riders (all *p*s < 0.001). The main effect of target showed that children punished free riders more than both self-reliant individuals (*p* < 0.001) and cooperators (*p* < 0.001). Additionally, neither the main effect of age (F_(2, 138)_ = 2.61, *p* = 0.08, η^2^_partial_ = 0.04), nor the Age × Target × Resource Endowment interaction (F_(3.82, 263.26)_ = 0.48, *p* = 0.74, η^2^_partial_ = 0.01), was significant.

As a robustness check, we conducted linear mixed-effects models using the same predictors, treating age as a continuous predictor and subject as a random effect. The same analyses were also conducted for evaluation and partner choice. These analyses yielded results consistent with the primary ANOVA findings, with detailed information provided in the Supplementary Materials (see “Linear Mixed-Effects Models”).

### 3.2. Evaluation

**Figure 3:**
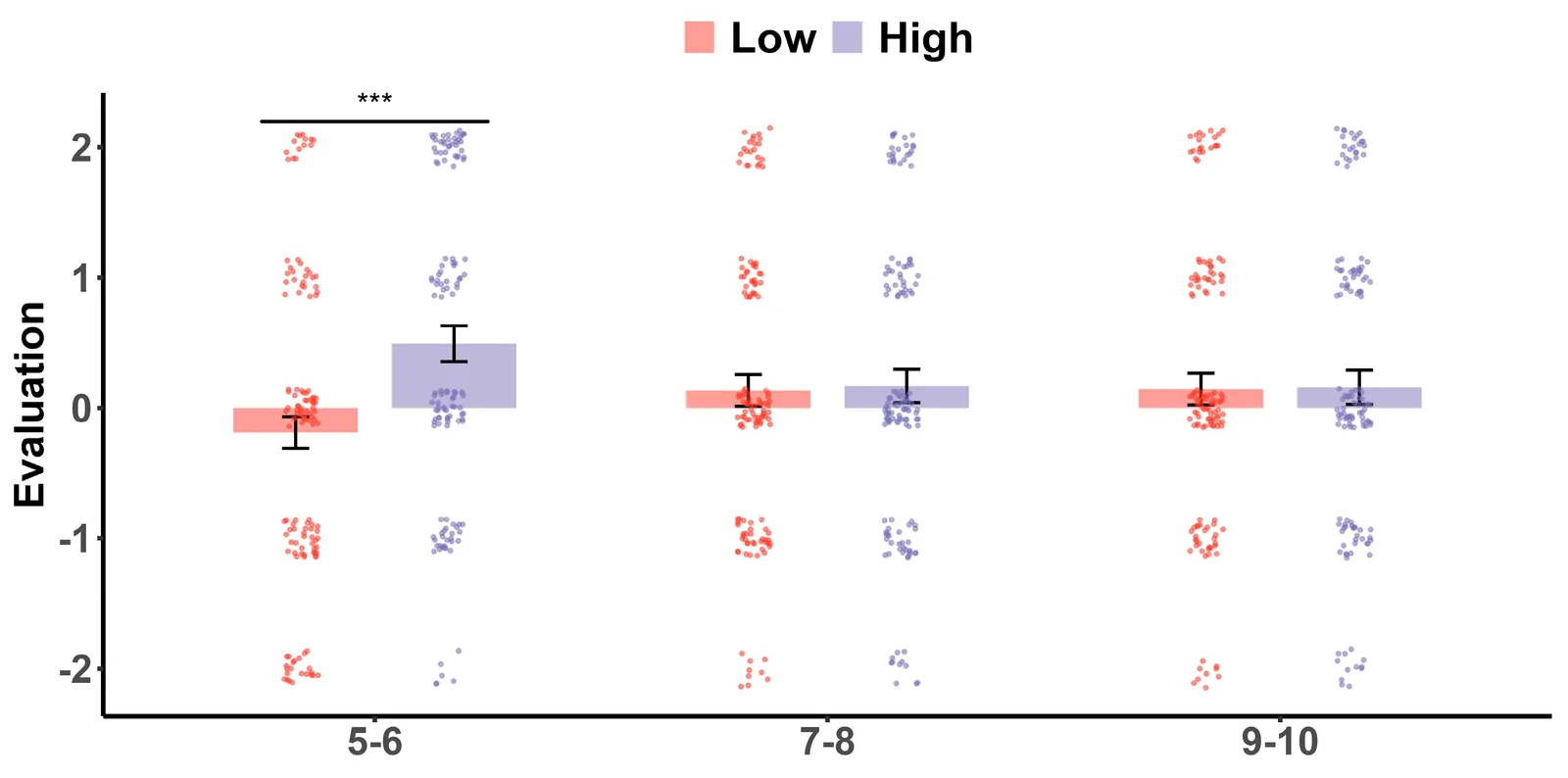
Interaction effects of different age and resource endowment on children’s evaluation patterns. Error bars represent the ±1 standard error of the mean. *** *p* < 0.001. Scatter points represent the data of each child with jitters. Asterisks indicate significant differences between the high- and low-resource-endowment conditions within each age group.

**Figure 4:**
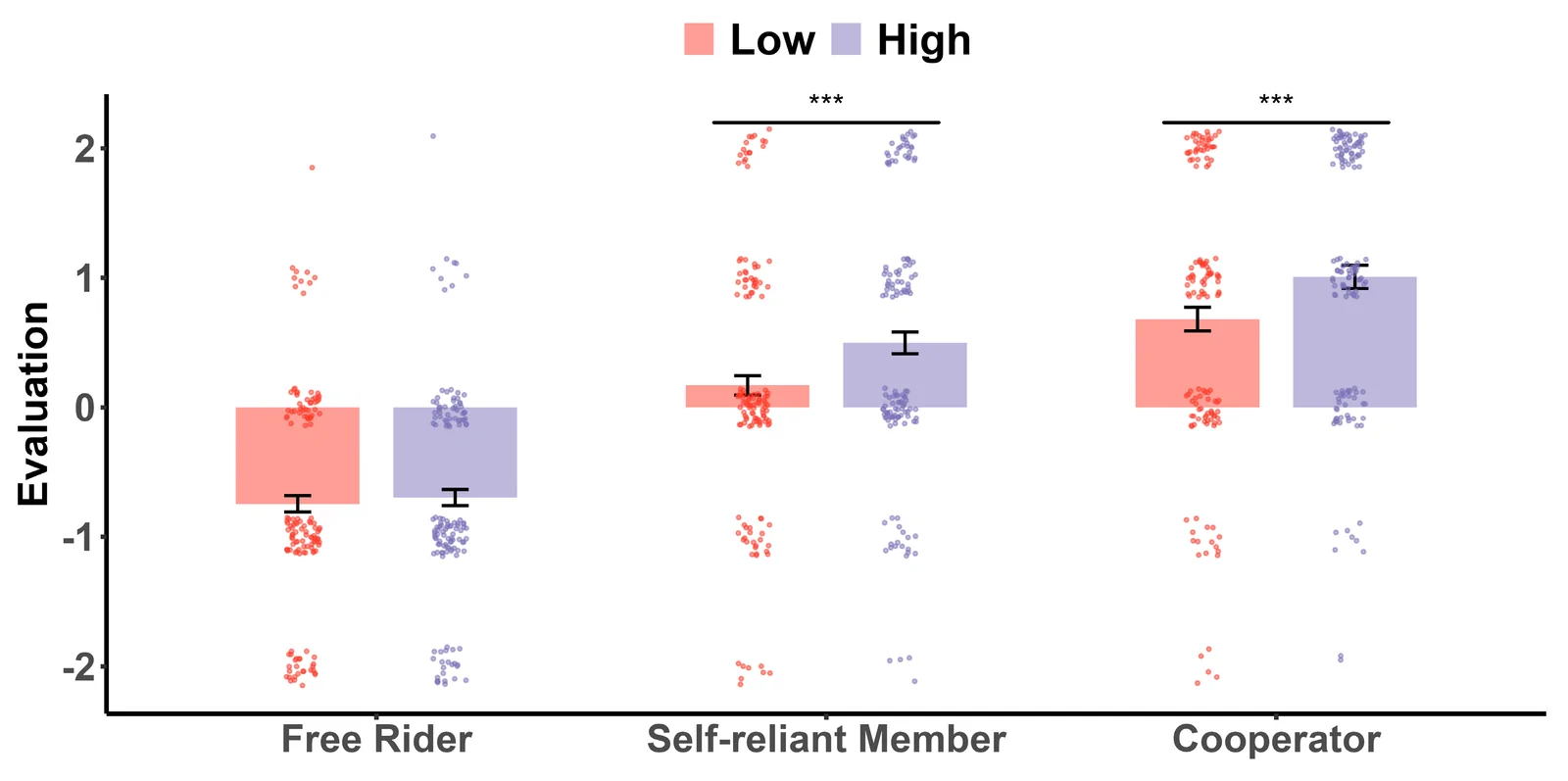
Interaction effects of different targets and resource endowment on children’s evaluation patterns. Error bars represent the ±1 standard error of the mean. *** *p* < 0.001. Scatter points represent the data of each child with jitters. Asterisks indicate significant differences between the high- and low-resource-endowment conditions within each target type.

There was a significant interaction between age and resource endowment (Figure 3), F_(2, 138)_ = 18.34, *p* < 0.001, η^2^_partial_ = 0.21. Children aged 5–6 evaluated targets with high resource endowment (Mean = 0.46, 95% CI = [0.31, 0.61]) more positively than targets with low resource endowment (Mean = −0.22, 95% CI = [−0.40, −0.04]), but the other age groups showed no significant difference in their evaluations (7–8, High: Mean = 0.17, 95% CI = [0.02, 0.32]; 7–8, Low: Mean = 0.13, 95% CI = [−0.04, 0.31]; 9–10, High: Mean = 0.19, 95% CI = [0.05, 0.34]; 9–10, Low: Mean = 0.18, 95% CI = [0.01, 0.35]). There was also a target × resource endowment interaction (Figure 4), F_(2, 275.46)_ = 4.92, *p* < 0.01, η^2^_partial_ = 0.03. Children evaluated self-reliant members with high resource endowment (Mean = 0.51, 95% CI = [0.34, 0.67]) more positively than self-reliant members with low resource endowment (Mean = 0.17, 95% CI = [−0.003, 0.34]). Similarly, children evaluated cooperators with high resource endowment (Mean = 1.01, 95% CI = [0.84, 1.17]) more positively than cooperators with low resource endowment (Mean = 0.67, 95% CI = [0.50, 0.84]). However, children evaluated free riders similarly (High: Mean = −0.69, 95% CI = [−0.83, −0.55]; Low: Mean = −0.75, 95% CI = [−0.89, −0.61]), regardless of resource endowment. There was also a main effect on resource endowment, F_(1, 138)_ = 23.11, *p* < 0.001, η^2^_partial_ = 0.14, such that children evaluated targets with high resource endowment (Mean = 0.27, 95% CI = [0.19, 0.36]) more positively than targets with low resource endowment (Mean = 0.03, 95% CI = [−0.07, 0.13]). Hence, the results for evaluation mirrored those of punishment.

There was also a significant interaction between age and target (F_(3.43, 236.46)_ = 4.43, *p* < 0.01, η^2^_partial_ = 0.06) and a significant main effect of target (F_(1.71, 236.46)_ = 126.94, *p* < 0.001, η^2^_partial_ = 0.48). Briefly, the interaction findings showed that children aged 5–6 did not significantly differ in their evaluation of self-reliant individuals and cooperators (*p* = 0.99); however, children aged 7–8 (*p* < 0.01) and 9–10 (*p* < 0.001) evaluated self-reliant individuals significantly more negatively than cooperators. Moreover, children aged 5–6, 7–8, and 9–10 evaluated self-reliant individuals or cooperators significantly more positively than free riders (all *p*s < 0.001). The main effect of target showed that free riders were evaluated most negatively, self-reliant individuals received intermediate evaluations, and cooperators were evaluated most positively (all *p*s < 0.001). Additionally, neither the main effect of age (F_(2, 138)_ = 0.24, *p* = 0.79, η^2^_partial_ = 0.003), nor the Age × Target × Resource Endowment interaction (F_(3.99, 275.46)_ = 1.22, *p* = 0.30, η^2^_partial_ = 0.02), was significant.

### 3.3. Partner Choice

**Figure 5:**
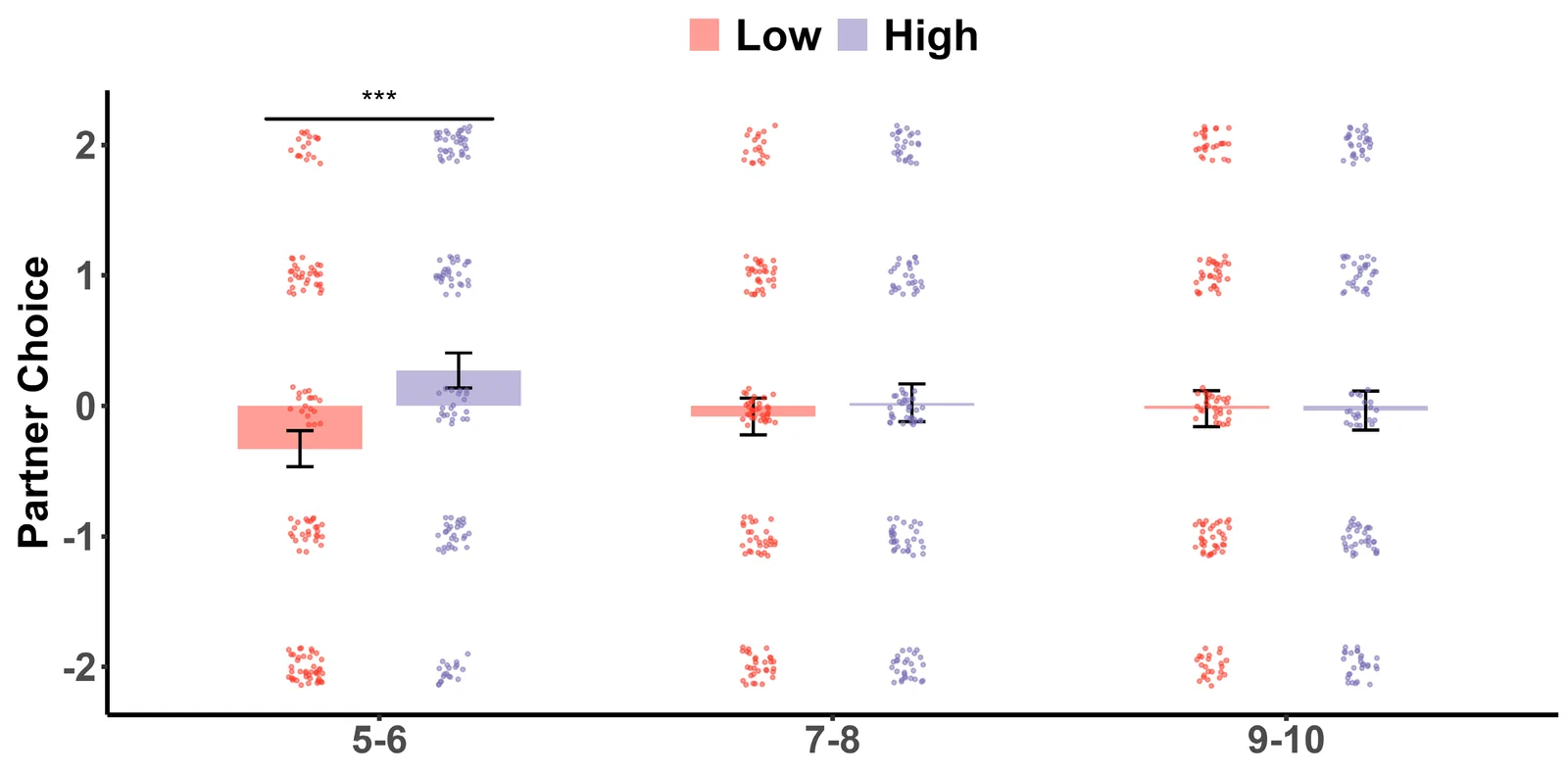
Interaction effects of different age and resource endowment on children’s partner choice patterns. Error bars represent the ±1 standard error of the mean. *** *p* < 0.001. Scatter points represent the data of each child with jitters. Asterisks indicate significant differences between the high- and low-resource-endowment conditions within each age group.

**Figure 6:**
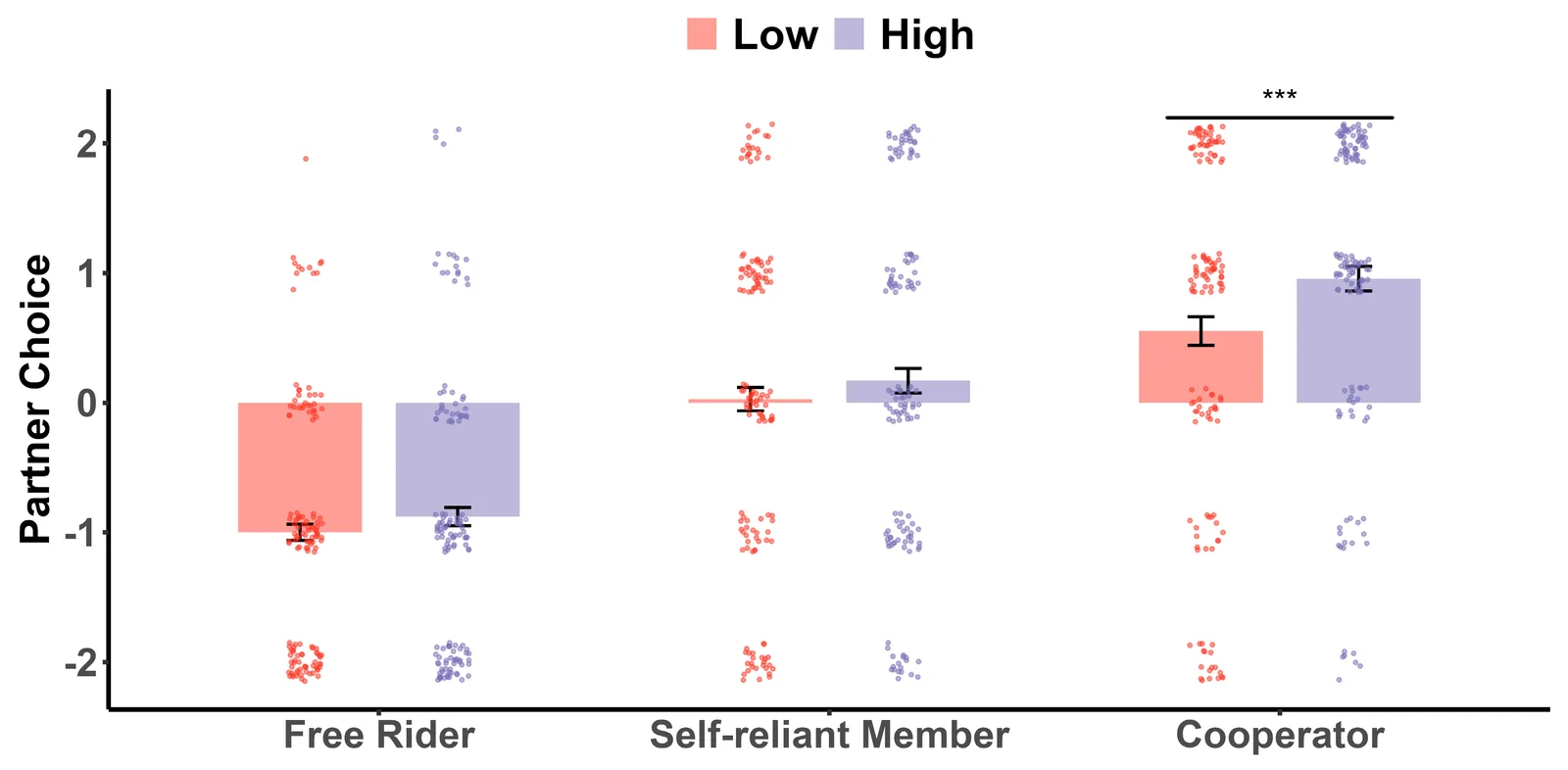
Interaction effects of different targets and resource endowment on children’s partner choice patterns. Error bars represent the ±1 standard error of the mean. *** *p* < 0.001. Scatter points represent the data of each child with jitters. Asterisks indicate significant differences between the high- and low-resource-endowment conditions within each target type.

There was a significant interaction between age and resource endowment (Figure 5), F_(2, 138)_ = 12.49, *p* < 0.001, η^2^_partial_ = 0.15. Children aged 5–6 preferred targets with high resource endowment as partners (Mean = 0.28, 95% CI = [0.04, 0.52]) compared to targets with low resource endowment (Mean = −0.32, 95% CI = [−0.56, −0.08]), while children aged 7–8 (High: Mean = −0.04, 95% CI = [−0.27, 0.20]; Low: Mean = −0.14, 95% CI = [−0.37, 0.09]) and 9–10 (High: Mean = 0.01, 95% CI = [−0.22, 0.25]; Low: Mean = 0.03, 95% CI = [−0.20, 0.26]) did not show a significant difference in preferences between targets with different resource endowment. There was a significant target × resource endowment interaction (Figure 6), F_(1.92, 265.52)_ = 3.47, *p* < 0.05, η^2^_partial_ = 0.03. When choosing partners, children did not exhibit a significant difference in free riders (High: Mean = −0.87, 95% CI = [−1.05, −0.70]; Low: Mean = −1.00, 95% CI = [−1.16, −0.84]) or self-reliant members (High: Mean = 0.18, 95% CI = [−0.04, 0.40]; Low: Mean = 0.03, 95% CI = [−0.19, 0.25]) across levels of resource endowment, but were more likely to choose cooperators with high resource endowment (Mean = 0.95, 95% CI = [0.76, 1.14]) as partners compared to cooperators with low resource endowment (Mean = 0.54, 95% CI = [0.33, 0.76]). Similarly, there was a main effect of resource endowment, F_(1, 138)_ = 19.08, *p* < 0.001, η^2^_partial_ = 0.12, as children showed a stronger preference for choosing targets with high resource endowment as partners (Mean = 0.09, 95% CI = [−0.05, 0.22]) than targets with low resource endowment (Mean = −0.14, 95% CI = [−0.28, −0.008]). Hence, the results for peer choice were also broadly consistent with the punishment patterns, though there were minor differences in the interaction effect between target and resource endowment regarding self-reliant members.

There was also a significant interaction between age and target (F_(3.18, 219.38)_ = 7.77, *p* < 0.001, η^2^_partial_ = 0.10) and a significant main effect of target (F_(1.59, 219.38)_ = 115.92, *p* < 0.001, η^2^_partial_ = 0.46). Briefly, the interaction findings showed that children aged 5–6 did not significantly differ in their partner preferences of self-reliant individuals and cooperators (*p* = 0.71); however, children aged 7–8 (*p* < 0.01) and 9–10 (*p* < 0.001) were significantly less likely to choose self-reliant individuals than cooperators as partners. Moreover, children aged 5–6, 7–8, and 9–10 were significantly more likely to choose self-reliant individuals or cooperators than free riders as partners (all *p*s < 0.001). The main effect of target showed that children were least willing to choose free riders as partners, showed intermediate willingness to choose self-reliant individuals, and were most willing to choose cooperators, with all pairwise comparisons reaching significance (all *p*s < 0.001). Additionally, neither the main effect of age (F_(2, 138)_ = 0.25, *p* = 0.78, η^2^_partial_ = 0.004), nor the Age × Target × Resource Endowment interaction (F_(3.85, 265.52)_ = 0.83, *p* = 0.50, η^2^_partial_ = 0.01), was significant.

## 4. Discussion

Our study investigated how children incorporate resource endowment as a contextual cue in making third-party responses in collective games. The results generally supported our hypotheses and revealed several main findings.

Firstly, children displayed a resource-based preference by favoring individuals with greater resource endowment. This trend was most pronounced in younger children (age 5–6). Such a preference may help dependent children secure access to vital resources for survival (Kenward et al., 2015). Further, Ahl et al. (2019) showed that children could use real-world wealth as resource cues to infer social status and competence. Our findings support and extend this work by showing that children are also sensitive to differences in task-specific resources, such as token endowments, even when these tokens have no explicit real-world meaning. This suggests that children’s sensitivity to resource differences is not limited to familiar material possessions, highlighting the robustness of resource-based preferences across different forms of resource cues.

Secondly, we observed an age-related pattern in children’s responses to resource endowment. Children aged 5–6 years showed a robust preference for high-endowment individuals, whereas comparable preferences were not statistically significant among children aged 7–8 or 9–10 years. Moreover, the age-related decline in resource-based preference we found is consistent with results of other studies (Shu et al., 2024; X. Yang & Dunham, 2022). One possible inference is that, as children’s cognitive abilities develop and social experiences accumulate, they may become more sensitive to the multifaceted origins of resource distribution and learn to dissociate material wealth from personal worth. Accordingly, one speculative possibility is that older children may place relatively greater weight on inferred internal traits, such as perceived cooperative intent, and less weight on external indicators such as resource possession when forming social responses. More broadly, this pattern may reflect a developmental shift from an emphasis on resource-related cues toward judgments that are increasingly informed by the values of children’s sociocultural environment. However, these inferences were not directly measured in the present study, and further research is needed to examine the processes underlying this age-related pattern.

More importantly, our findings showed that from age five, children can differentiate between free riders and self-reliant individuals and exhibit different responses. Across all age groups, children consistently showed more negative responses toward free riders than cooperators and self-reliant individuals, regardless of their resource endowment. These findings align with prior evidence that children are sensitive to fairness violations and are motivated to uphold fairness through social intervention (F. Yang et al., 2018). In contrast, their responses to self-reliant individuals are more nuanced, with children being more likely to justify self-reliance when the individual possesses abundant resources.

The distinction likely arises from fundamental differences between the two strategies (free-riding and self-reliance) in their exploitative intent and the extent of fairness violation (Gross & De Dreu, 2019; Gross et al., 2020). Crucially, we cautiously speculate that children’s differentiated responses to these behaviors may reflect an emerging form of social competence. Whereas free-riding elicited consistently negative responses regardless of resource endowment, responses to self-reliance were more context-dependent. This pattern suggests that children flexibly adjust their responses based on the target’s social strategy and resource, indicating an early form of context-sensitive social responses (Blake et al., 2015; Engelmann & Tomasello, 2019; Kenward & Dahl, 2011). If so, such increasing competence may enable children to respond to complex social situations in a more appropriate manner. In addition, one tentative inference is that children’s greater tolerance of self-reliance among resource-rich individuals may shape the norms surrounding self-reliance. If so, by granting greater tolerance to these individuals, children may promote a social context in which self-reliance is viewed as acceptable, expected, or even valued among those with greater resources. Future research could examine whether such tolerance contributes to the prevalence of self-reliance among well-resourced individuals and whether, in turn, this pattern is associated with greater social asymmetries or disadvantages for more dependent populations (Gross et al., 2020; Piketty & Saez, 2014; Saez & Zucman, 2016). Thus, the present findings provide a basis for investigating how children’s context-sensitive social responses may contribute to social norms and patterns over time.

Several limitations of the present research should be acknowledged. First, the present study relied on a simplified laboratory task involving fictional peers and equivalent payoffs, which may limit the ecological validity of the findings. Although this design allowed us to control relevant experimental factors, children’s responses in such scenarios may not fully reflect how they judge non-cooperative strategies and resource differences in real-world social interactions. In addition, token endowment was represented by only two discrete levels, which further simplified the complexity of real-world resource differences. Future research could adopt more ecologically valid designs to examine whether these patterns generalize to children’s responses in real-world social contexts.

Second, although resource endowment shaped children’s evaluations and punishment decisions toward self-reliant members, this effect did not extend to partner choice. This divergence across measures may reflect their different social functions. Unlike evaluation and punishment, partner choice concerns a prospective interaction that may have more direct consequences for children themselves. Hence, one speculative possibility is that, when selecting a future partner, children may place greater weight on the target’s behavioral strategy or inferred cooperative intent, and relatively less weight on resource endowment. Thus, the different pattern observed for partner choice may provide meaningful information about how children weigh social cues across distinct forms of social response, rather than simply reflecting inconsistency across measures. However, the present study cannot directly determine why these differences emerge, and future research should further examine the factors contributing to these patterns.

Third, the cultural and socioeconomic scope of the sample should also be considered. Participants were Han Chinese children attending public kindergartens or primary schools in Beijing and therefore primarily represented an urban, relatively socioeconomically advantaged population. Children’s third-party responses may vary across cultural and socioeconomic contexts. Future research should therefore examine whether the present patterns generalize to children from more diverse cultural and socioeconomic backgrounds.

Fourth, the present study employed a cross-sectional design. Thus, the observed differences across age groups cannot establish developmental changes over time. Longitudinal research is needed to examine how children’s weighting of behavioral strategies and resource information changes across development.

Additionally, because the six conditions were presented within subjects, repeated exposure to similar scenarios may have introduced order, fatigue, expectancy or carryover effects. Although condition order was counterbalanced to reduce potential impacts, such influences cannot be completely ruled out. Future studies could complement the present design with between-subject designs to examine the robustness of these findings.

A further limitation concerns how children interpreted self-reliance. In the present study, children observed a concrete individual-solution strategy in which the target used personal tokens to protect only their own resources. The present findings concern children’s responses to this observable behavior and therefore do not require children to distinguish self-reliance conceptually from independence, competence, selfishness, or simple non-cooperation. Nevertheless, these related interpretations may have contributed to their responses. More generally, because we did not collect explanations for children’s responses, we cannot determine whether their responses reflected moral reasoning, resource preferences, perceived competence, or other interpretations of the scenarios. Future research should directly examine how children interpret the different social strategies, as well as the reasons underlying their own responses. Specifically, one useful approach would be to directly assess theoretically relevant factors that may shape children’s responses, such as their inferences about agents’ intentions, characteristics and social norms (Peretz-Lange et al., 2022). Another useful approach would be to collect children’s verbal explanations for their choices, which may provide further insight into the reasoning processes underlying their responses and whether these processes differ across age.

Finally, individual differences in cognitive abilities may also contribute to children’s social responses. For example, executive functioning may support children’s ability to integrate multiple sources of information and regulate responses to salient cues, whereas Theory of Mind may influence how they infer the intentions and characteristics underlying others’ behavior. Because these abilities were not assessed in the present study, their contribution to the observed response patterns cannot be determined. Future research could directly measure executive functioning and Theory of Mind to examine whether they account for individual and age-related differences in children’s third-party responses.

To summarize, the present study highlights target-specific effects of resource endowment on children’s third-party responses in cooperative contexts. The findings suggest age-related differences in resource-based preferences and reveal distinctions in how children respond to self-reliant targets and free riders with varying levels of resource endowment. Specifically, we identify two developmental patterns in our sample: (1) a shift in resource-based preferences, whereby children aged 5–6 years tend to favor individuals with greater resource endowment, whereas this tendency diminishes with age, possibly reflecting a reduced reliance on resource cues and greater consideration of other social information, such as inferred cooperative intent; and (2) an emerging ability to flexibly adjust third-party responses according to social context, reflected in the integration of resource endowment and social strategy. Overall, these findings highlight the increasing sophistication of children’s social cognition and advance our understanding of third-party decision-making in cooperative contexts across early to middle childhood.

## Data Availability

The data and code that support the findings are available in Supplemental Materials.

## Supplementary Materials

The following supporting information can be downloaded at https://www.mdpi.com/article/10.3390/bs16081422/s1 (Amir et al., 2026; Bardach et al., 2025; House, 2018; Im et al., 2025; Killen & Smetana, 2015; Schlingloff-Nemecz et al., 2025; Thompson, 2006; Turiel, 2006; Warneken, 2018; F. Yang et al., 2018).
