# Supplementary material for: Developmental Patterns in Cooperative Contexts: How Children Integrate Social Strategy and Resource Endowment in Third-Party Responses
Source: Behav Sci (Basel). 2026 Aug 19;16(8):1422. doi: 10.3390/bs16081422 (PMC13509671; doi:10.3390/bs16081422)
Supplement: Supplementary file 1 [file behavsci-16-01422-s001.zip › BR Supplementary Material-revised.pdf]

Supplementary Material

Supplementary Methods

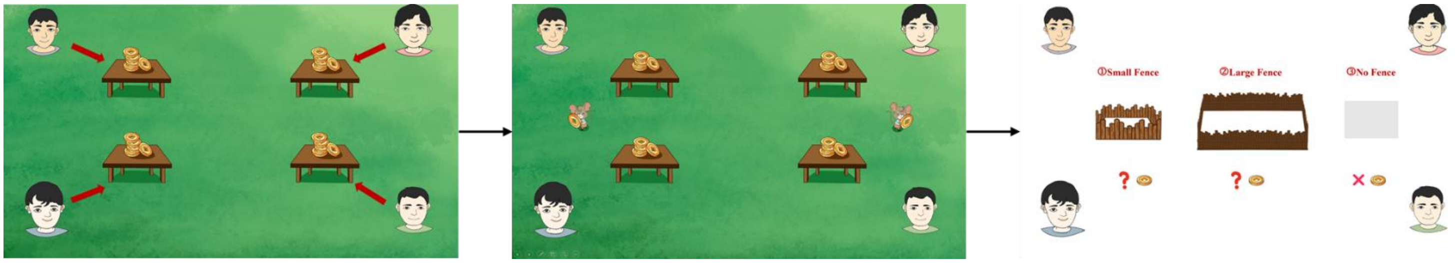

Figure S1. Key Rules of the Collective Dilemma Game. The figure displays four cartoon avatars of boys. Avatars were randomly selected from an avatar library and gender-matched to the child participants.

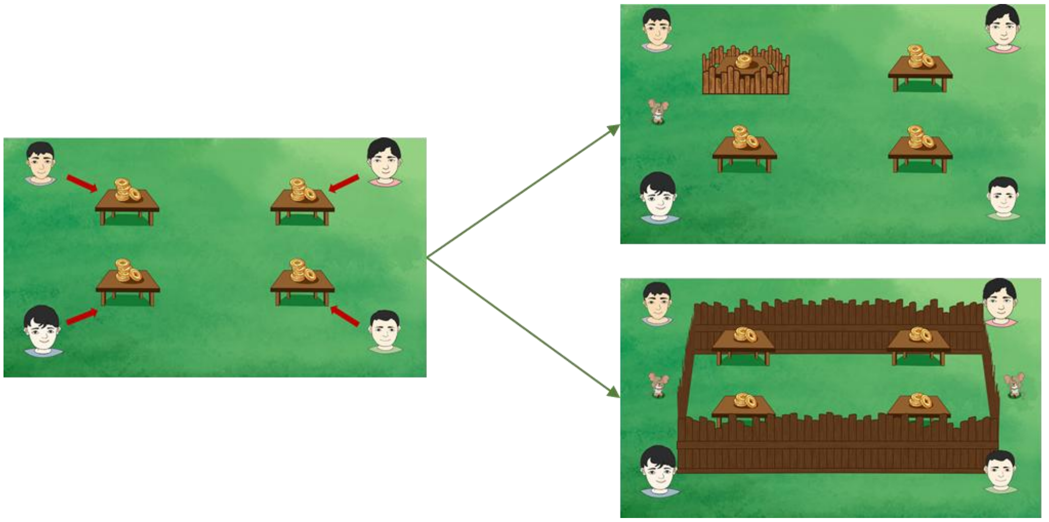

Figure S2. Two Possible Strategies in the Collective Dilemma Game: constructing a small fence (top right) or a large fence (bottom right).

Age Grouping

We grouped children into three age bands (5–6, 7–8, and 9–10 years) to capture potential non-linear or stage-related transitions in social responses. Developmental research suggests that children aged 5–6 are typically in the preschool period, during which social cognition is still emerging. At this stage, children's understanding of social norms is relatively limited, and their behavior is often more directly driven by situational cues than by stable internalized standards (Thompson, 2006; Warneken, 2018). By contrast, children aged 7–8 and 9–10 enter formal schooling and increasingly participate in more complex social interaction. During this period, they acquire cooperative rules and social evaluative standard (Bardach et al., 2025). In particular, ages 7–8 have been identified as a critical period for advances in social-cognitive abilities and the internalization of social norms (Amir et al., 2026; House, 2018; Im et al., 2025; Killen & Smetana, 2015). Children at this stage begin to demonstrate more stable understanding of abstract social concepts and apply them flexibly across contexts (Schlingloff-Nemecz et al., 2025; Turiel, 2006; Yang et al., 2018). As children approach 9–10 years, continued cognitive maturation and accumulated social experience further refine their social reasoning. Overall, this age grouping captures meaningful developmental transitions in social cognition and provides a theoretically grounded framework for examining how children at different stages respond to non-cooperators.

Moreover, modeling age as a continuous predictor typically assumes a linear (or smoothly parametric) relationship between age and the outcome. However, developmental processes—particularly those involving third-party responses towards self-reliance—may involve non-linear shifts rather than smooth age-related increases. Grouping children into two-year bands allows for the detection of potential non-linear or stage-related changes in behavior.

Table S1

Table S1. Descriptive Statistics for Punishment, Evaluation, and Partner Choice Across Experimental Conditions

Age	Target	Resource	Punishment	Evaluation	Partner Choice
**5-6**	Free Rider	Low	2.00 (1.40)	-0.93 (0.86)	-1.11 (0.96)
	Free Rider	High	1.58 (1.39)	-0.53 (0.92)	-0.53 (1.22)
	Self-reliant Member	Low	1.07 (1.34)	0.16 (1.26)	0.24 (1.45)
	Self-reliant Member	High	0.56 (1.03)	0.96 (1.09)	0.71 (1.44)
	Cooperator	Low	1.33 (1.33)	0.11 (1.15)	-0.09 (1.55)
	Cooperator	High	0.58 (0.97)	0.96 (1.00)	0.67 (1.26)
**7-8**	Free Rider	Low	1.35 (1.33)	-0.60 (0.87)	-1.04 (0.90)
	Free Rider	High	1.48 (1.44)	-0.65 (0.76)	-0.98 (0.96)
	Self-reliant Member	Low	0.79 (1.15)	0.15 (1.01)	-0.08 (1.33)
	Self-reliant Member	High	0.67 (1.06)	0.33 (1.06)	0.00 (1.20)
	Cooperator	Low	0.67 (1.12)	0.85 (1.03)	0.71 (1.11)
	Cooperator	High	0.54 (1.03)	0.81 (1.12)	0.88 (1.30)
**9-10**	Free Rider	Low	1.40 (1.32)	-0.71 (0.85)	-0.85 (1.05)
	Free Rider	High	1.65 (1.39)	-0.90 (0.81)	-1.10 (1.02)
	Self-reliant Member	Low	0.62 (1.10)	0.21 (0.80)	-0.06 (1.17)
	Self-reliant Member	High	0.54 (1.05)	0.23 (0.83)	-0.17 (1.24)
	Cooperator	Low	0.19 (0.57)	1.04 (0.87)	1.00 (1.19)
	Cooperator	High	0.19 (0.67)	1.25 (0.76)	1.31 (0.83)

Note. Values are presented as Mean (SD).

Linear Mixed-Effects Models

As a robustness check, we fitted separate linear mixed-effects models for punishment, evaluation, and partner choice, treating children's exact age as a continuous predictor. Each model included age, target strategy, resource endowment, and their interactions as fixed effects. Participant-specific random intercepts were included to account for individual differences in overall response levels. Because each child responded under all target-strategy and resource-endowment conditions, the effects of these two within-participant factors could also vary across children. We therefore included random slopes for target strategy and resource endowment, allowing the model to capture individual differences in children's sensitivity to these experimental manipulations. All analyses were conducted in R 4.5.1, with linear mixed-effects models fitted using the lme4 and lmerTest packages and follow-up comparisons conducted using the emmeans package.

Significant Age × Resource Endowment interactions were consistently observed for punishment, $F_{(1, 139)} = 19.52$, $p < 0.001$, evaluation, $F_{(1, 139)} = 20.47$, $p < 0.001$, and partner choice, $F_{(1, 139)} = 21.81$, $p < 0.001$. Follow-up analyses showed that the age slopes differed significantly between the low- and high-resource conditions for punishment ($b = -0.13$, $t = -4.42$, $p < 0.001$), evaluation ($b = 0.15$, $t = 4.52$, $p < 0.001$), and partner choice ($b = 0.15$, $t = 4.67$, $p < 0.001$). Specifically, punishment showed a more negative age-related trend in the low- than in the high-resource condition, whereas evaluation and partner choice showed more positive age-related trends in the low- than in the high-resource condition. Together, the directions of the model-predicted trends were consistent with an age-related reduction in resource-based preference.

Significant Target × Resource Endowment interactions were also found for punishment, $F_{(2, 278)} = 4.12$, $p < 0.05$, evaluation, $F_{(2, 278)} = 4.78$, $p < 0.01$, and partner choice, $F_{(2, 278)} = 3.50$, p

< 0.05. Follow-up comparisons at the mean age showed that resource endowment did not significantly affect punishment of free riders ($b = 0.01$, $t = 0.09$, $p = 0.93$), whereas punishment was higher in the low- than in the high-resource condition for self-reliant members ($b = 0.23$, $t = 3.04$, $p < 0.01$) and cooperators ($b = 0.28$, $t = 3.68$, $p < 0.001$). For evaluation, no significant resource difference was observed for free riders ($b = -0.05$, $t = -0.62$, $p = 0.53$), whereas self-reliant members and cooperators were evaluated more positively in the high- than in the low-resource condition (both bs = −0.33, ts = −4.09, ps < 0.001). For partner choice, the resource difference was not significant for free riders ($b = -0.12$, $t = -1.39$, $p = 0.17$) or self-reliant members ($b = -0.14$, $t = -1.63$, $p = 0.10$), whereas cooperators were preferred more strongly in the high- than in the low-resource condition ($b = -0.40$, $t = -4.65$, $p < 0.001$).

Age × Target interactions were also significant for punishment, $F_{(2, 139)} = 3.86$, $p < 0.05$, evaluation, $F_{(2, 139)} = 4.90$, $p < 0.01$, and partner choice, $F_{(2, 139)} = 8.26$, $p < 0.001$. In contrast, the Age × Target × Resource Endowment interaction was not significant for punishment, $F_{(2, 278)} = 0.76$, $p = 0.47$, evaluation, $F_{(2, 278)} = 0.35$, $p = 0.71$, or partner choice, $F_{(2, 278)} = 0.83$, $p = 0.44$. Significant main effects of Target and Resource Endowment were also observed across all three outcomes, whereas the main effect of age was significant only for punishment, $F(1, 139) = 4.54$, $p = .035$.

Overall, the mixed-effects analyses using exact age as a continuous predictor provided converging evidence for the principal Age × Resource Endowment and Target × Resource Endowment patterns identified in the primary analyses with ANOVA.

To facilitate interpretation of the interaction patterns, we plotted population-level predictions from the full linear mixed-effects models (Figure S3). Within each target-strategy panel, differences in the slopes of the low- and high-resource lines illustrate the Age × Resource

Endowment interaction, whereas differences in the separation between these lines across target strategies illustrate the Target × Resource Endowment interaction.

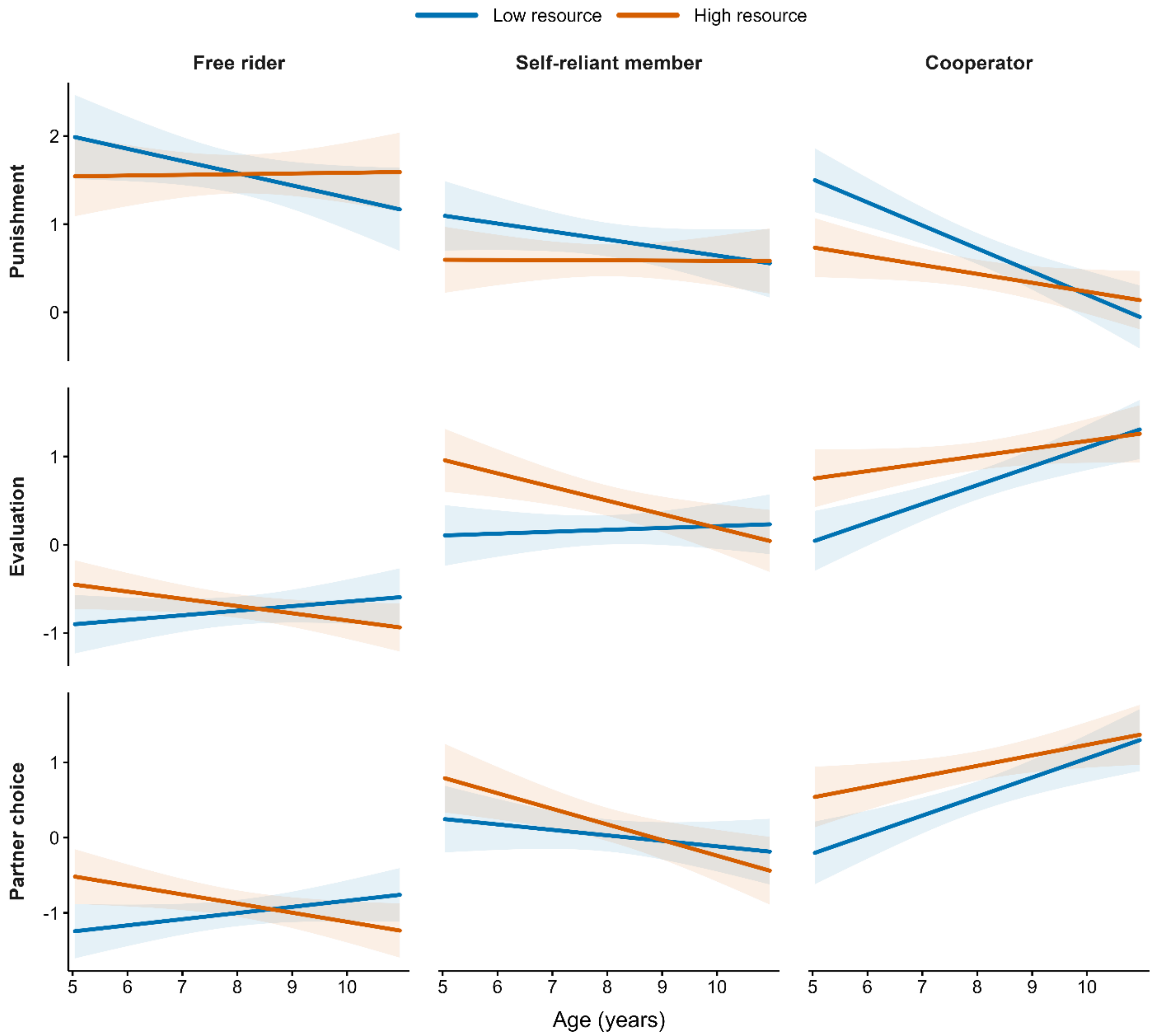

Figure S3. Model-predicted punishment, evaluation, and partner choice as a function of continuous age, target strategy, and resource endowment. Lines represent population-level predictions from the linear mixed-effects models, and shaded areas indicate 95% confidence intervals.

References

Amir, D., Ahl, R. E., Jordan, M. R., Bolotin, H., Bogese, M., González, G. T., Callaghan, T., Sugiyama, L. S., Otali, E., Tusiime, P., Bangayan, S., Snodgrass, J. J., & McAuliffe, K. (2026). The emergence of cooperative behaviors, norms, and strategies across five diverse societies. *Science Advances*, *12*(6), eadw9995. https://doi.org/10.1126/sciadv.adw9995

Bardach, L., Kalinowski, R., & Bailey, D. H. (2025). Differentiation in Cognitive Abilities Beyond g: The Emergence of Domain-Specific Variance in Childhood. *Psychological Science*, *36*(3), 168-183. https://doi.org/10.1177/09567976251321382

House, B. R. (2018). How do social norms influence prosocial development? *Current Opinion in Psychology*, *20*, 87-91. https://doi.org/10.1016/j.copsyc.2017.08.011

Im, E. J., Shirahatti, A., & Isik, L. (2025). Early neural development of social interaction perception: evidence from voxel-wise encoding in young children and adults. *The Journal of Neuroscience*, *45*(1). https://doi.org/10.1523/JNEUROSCI.2284-23.2024

Killen, M., & Smetana, J. G. (2015). Origins and development of morality. In *Handbook of child psychology and developmental science: Socioemotional processes* (7th ed., pp. 701–749). John Wiley & Sons, Inc.. https://doi.org/10.1002/9781118963418.childpsy317

Schlingloff-Nemecz, L., Stavans, M., Revencu, B., Hashiya, K., Kobayashi, H., & Csibra, G. (2025). Children's Trait Inference and Partner Choice in a Cooperative Game. *Child Development*, *96*(4), 1458-1473. https://doi.org/10.1111/cdev.14247

Thompson, R. A. (2006). The Development of the Person: Social Understanding, Relationships, Conscience, Self. In *Handbook of child psychology: Social, emotional, and personality development* (6th ed., pp. 24–98). John Wiley & Sons, Inc..

Turiel, E. (2006). Thought, emotions, and social interactional processes in moral development. In *Handbook of moral development.* (pp. 7-35). Lawrence Erlbaum Associates Publishers.

Warneken, F. (2018). How Children Solve the Two Challenges of Cooperation. *Annual Review of Psychology*, *69*(1), 205-229. https://doi.org/10.1146/annurev-psych-122216-011813

Yang, F., Choi, Y.-J., Misch, A., Yang, X., & Dunham, Y. (2018). In Defense of the Commons: Young Children Negatively Evaluate and Sanction Free Riders. *Psychological Science*, *29*(10), 1598-1611. https://doi.org/10.1177/0956797618779061
